# Facing the pandemic and lockdown: an insight on mental health from a longitudinal study using diaries

**DOI:** 10.1038/s41537-022-00222-2

**Published:** 2022-03-15

**Authors:** Amaury C. Mengin, Melissa C. Allé, Estelle Koning, Bichthuy Pham, Sohee Park, Fabrice Berna, Anne Giersch

**Affiliations:** 1 University of Strasbourg, INSERM U1114, University Hospital of Strasbourg, 1, place de l’Hôpital, 67000 Strasbourg, France; 2 Great East Regional Psychotrauma Center, University Hospital of Strasbourg, Strasbourg, France; 3 Univ. Lille, CNRS, UMR 9193 - SCALab - Sciences Cognitives et Sciences Affectives, F-59000 Lille, France; 4 Department of Psychology, Vanderbilt University, Nashville, TN USA

**Keywords:** Psychosis, Schizophrenia

## Abstract

We conducted a longitudinal online study to examine attenuated psychotic symptoms (APS) over time in a sample of locked-down individuals. We used (i) questionnaires and (ii) the automatic analysis of the emotional content of narratives. Participants (*N* = 162) were recruited to complete an online survey 4 times between March and June 2020 (T1, T2, T3, T4). T1 completion coincided with the beginning of the lockdown, and T4 with the pandemic trough. Depression, anxiety, and stress were assessed with the DASS-42 and APS with the PQ-16. Psychosocial data such as the feeling of loneliness and social network size were also collected. The participants wrote daily narratives during the lockdown period. Anxiety and APS were the highest at T1 and decreased over time. APS and APS-associated distress were correlated with the DASS-42 at all times. APS arose acutely at the beginning of the pandemic, despite participants being socio-economically advantaged, and were related with negative emotions.

## Introduction

The COVID-19 outbreak, and associated lockdowns, affected populations worldwide and marked an unprecedented rupture with the daily life. Individual reactions to these society-level disruptions can teach us a lot about the psychological impact of the pandemic itself, as well as the mitigation strategies such as the lockdowns and how we adapt to these changes. To examine and document mental health during the lockdown, we conducted a longitudinal online study consisting of self-report questionnaires and personal narratives in the general community. We focused especially on the emergence of attenuated symptoms of psychosis (APS), which have been rarely explored in the general community, despite the known association between loneliness, stress, and psychosis. In the absence of face-to-face interviews, personal narratives were used to complete the evaluation of the participants’ mental states, especially their emotional states, considering the well-known association between negative emotions and psychotic symptoms^1^.

Numerous studies have investigated the psychological impact of the COVID-19 pandemic and lockdown around the world. Most studies recognized a highly negative psychological impact during the first weeks of lockdown, both in February in China and in March 2020 in the rest of the world^2–9^. Anxiety levels rose during the early days of lockdown^7^. However, results concerning the mental health as the pandemic settled in for a long haul from spring to summer 2020 suggest an evolving picture. Concerning depression, Cecchini et al.^3^ observed that depression levels increased from March 18 to April 17 in 595 Spanish adults^3^. Probst et al. noticed the same increase between April and July 2020 in 445 Austrian adults^10^. In contrast, Fancourt et al. showed a decrease in depression levels in 36,000 UK adults between March and August 2020^11^. A meta-analysis found small effects from pandemic lockdowns on anxiety and depression (Hedges’ *g* = 0.18 and 0.16, respectively)^12^. These findings suggest that the psychological outcomes during the pandemic evolve dynamically over time. However, how exactly psychosis risk dynamically evolves is especially unclear.

There were robust reasons to posit that the pandemic context would produce APS. First, the link between isolation and psychosis is supported by past research. Hallucinations and paranoia are fostered by isolation - such as during imprisonment, polar or space explorations^13^. As sensory deprivation is known to induce hallucinations^14^, Hoffman et al.^15^ suggested that social deprivation may have the same effect as sensory deprivation. Since then, a number of studies have confirmed a link between psychosis and loneliness^16,17^. Secondly, the link between stress and psychosis is well established. Stress is known to be a precipitating factor to psychosis in vulnerable individuals^18,19^. Negative affect and affective disturbances have also been associated with psychosis^20,21^. Klippel et al.^22^ suggested that the impact of stress on psychosis was mediated by negative affect. The pandemic-induced stress and negative affect may play a central role in increasing the psychosis risk. There have been a small number of studies that examined psychotic symptoms in the general population during COVID-19 lockdown. Castellini et al.^23^ found that interpersonal sensitivity and paranoid ideation decreased during the first weeks of lockdown in 130 Italian adults. Similarly, Tso & Park^24^ reported that an astonishing 65.6% of the 432 adults surveyed in Hong Kong reported clinical levels of depression, anxiety, and/or stress while 22.5% were showing signs of attenuated psychosis-like symptoms during the spring of 2020. In South Korea even though only localized lockdowns were enacted, elevated psychosis risk was observed in 12.8% of the 400 adult respondents^25^. These represent a drastic increase above the base rate prior to the pandemic. On the other hand, Bortolon et al.^26^ found that paranoia and hallucinations levels were relatively low in French general population^26^, but this study took place at a distance from the beginning of the lockdown period.

The present study longitudinally explored psychological distress among participants by asking them to answer online questionnaires on four successive time points and to fill in daily narratives, from the beginning of the first lockdown period in France to the period following lockdown and the first pandemic wave (in June 2020, which was thus considered as the baseline). First, we examined the evolution of stress, anxiety, depression, and attenuated psychotic symptoms over time. We hypothesized that psychological distress would be already high at the beginning of lockdown, i.e., our first measure, as it was demonstrated in numerous studies^9,27–32^. We expected a high level of distress at T1, but a progressive return to baseline levels of symptoms at a distance from lockdown. Whether this pattern would also be reflected in APS was unknown. Given the use of online questionnaires among the general community, it was important to verify whether we could replicate the relationship between negative emotions and psychosis risk. Therefore, we investigated how the evolution of symptoms might be associated with sociodemographic characteristics, lockdown conditions, psychosocial data, and life narrative emotional content.

## Results

A total of 162 participants were recruited. Their sociodemographic characteristics and lockdown conditions are detailed in Table 1. They were asked to fill in questionnaires at four successive periods. T1 corresponded to the beginning of lockdown, but participants were asked to fill in the questionnaires by retrospectively referring to their pre-lockdown state. We will refer to T1 as the initial measure and as the beginning of lockdown in the remainder of the text. T2 corresponded to the middle of lockdown and participants were asked to evaluate their state during the beginning of the lockdown. T3 corresponded to the end of lockdown, and T4 to the period after lockdown, when the pandemic was considered resolved in France and Europe and when the media did not yet speak about the possibility of a second wave. Therefore, scores at T4 are considered as reflecting the baseline condition of our participants regarding their mental health while scores at T1 and T2 reflected the effect of the stressful event represented by both the pandemic and lockdown.

**Table 1:** Sociodemographic characteristics and lockdown conditions of participants.

	*N* = 162	(%)
Mean age (SD)	42.9 (14.8)	
Women	130	(80.2)
Men	32	(19.8)
Employed	140	(86.4)
Unemployed	22	(13.6)
Non-medical field	143	(88.3)
Medical field	19	(11.7)
Education		
High-school diploma	28	(17.3)
MSc	51	(31.5)
PhD	83	(51.2)
Family status		
Married or in a relationship	114	(70.4)
Single	48	(29.6)
*Lockdown conditions*	*N* = 134	
Housing		
Apartment	64	(47.8)
House	65	(48.5)
Apartment sharing	4	(3.0)
Student residence	1	(0.7)
Lockdown place		
At my home	114	(85.1)
Family	9	(6.7)
Friends	2	(1.5)
Elsewhere	9	(6.7)
Mean household surface in m^2^ (SD)	107 (56)	min = 25
		max = 300
Access to nature		
Yes	101	(75.4)
No	33	(24.6)
Teleworking		
Yes	109	(81.3)
No	25	(18.7)

### The number of participants meeting the APS cut-off score and of participants lost at follow-up

In addition to sociodemographic data, we measured Depression, Anxiety, and Stress Scale with the DASS-42. Attenuated psychotic symptoms were evaluated with the PQ-16, a screening tool with three subscales (unusual thought content, delusional ideas, and paranoia; perceptual abnormalities and hallucinations; negative symptoms)^33–35^. The UCLA Loneliness Scale^36^ was used to assess subjective feelings of loneliness and the Social Network Index (SNI)^37^ to quantify objective levels of social isolation by incorporating the diversity (i.e., number of social roles) and size (number of people with whom the respondent has regular contact in person or remotely) of social networks.

Thirty among the 162 participants reached the APS cut-off score on T1. Among those, only 12 (40%) then filled in all questionnaires, and 8 (26.7%) stopped after the first one. In contrast, among the 132 participants who did not reach the APS cut-off score on T1, 94 (71.2%) filled in all questionnaires and 19 (14.4%) stopped after the first one. Those proportions differ significantly: the rate of participants filling in all questionnaires (P-all) was much lower in participants who reached the APS cut-off score on T1 than in those scoring below the cut-off (40 vs. 71.2%, *χ*^2^(df=1) = 10.5, *p* < .005). These results suggest that participants lost at follow-up (P-lost) differ from P-all participants: a detailed comparison of P-lost and P-all participants can be found in Table 2. To ensure that the high initial scores of participants lost at follow-up did not induce an artificial decrease of symptoms over time, we analyzed separately the evolution of APS over time in the participants filling in 3 instead of 4 questionnaires. Twenty-one among the 55 P-lost participants filled in 3 questionnaires and among those 21 participants, 8 reached the APS cut-off score. Eight among 21 participants is a significantly higher rate (38.1%) than the rate observed in P-All participants (10.5%, *χ*^2^(df = 1)=9.6, *p* < 0.005). Symptoms decreased significantly in those 21 participants across the three measurement points (the total PQ-16 score averaged over the 21 participants decreased from 4.2 to 3 at T3, F[2, 38] = 4.4, *p* < 0.05, partial *η*^2^ = 0.19). Only 3 participants among those 21 increased their PQ-16 scores by 1 or 2.

**Table 2:** Sociodemographic, psychosocial and psychological characteristics of those who participated at all time points (P-All) and those lost to follow-up (P-Lost) at T1.

	P-All* (*n* = 107) (Mean ± SD)	P-Lost** (*n* = 55)(Mean ± SD)	Statistical comparison of the scores between groups
4/3/2/1 questionnaires filled in	107/0/0/0	0/21/7/27	
Age	43.5 ± 14.7	38.3 ± 13.9	*F* (1, 160) = 4, *p* < 0.05, *η*^2^ = 0.026
Sex (M/F)	19/88	14/41	χ^2^ n.s.
Education Level	16.9 ± 1.7	16.8 ± 1.5	n.s.
Household surface	107.7 ± 67.3	99.1 ± 50.9	n.s.
Level of concern about COVID-19	1.6 ± 0.8	1.6 ± 0.7	n.s.
DASS-42			
Depression	7.7 ± 8.5	6.9 ± 8	n.s.
Anxiety	5.2 ± 5.5	4.6 ± 6.1	n.s.
Stress	11.3 ± 8.3	10.3 ± 8.8	n.s.
Total	24.1 ± 19.8	21.8 ± 20.3	n.s.
Loneliness (UCLA Loneliness Scale)	34 ± 10.4	35.5 ± 10.5	n.s.
Social contacts (Social Network Index)			
Total number	17.5 ± 7.9	17.1 ± 8.5	n.s.
Diversity	5.6 ± 1.7	5.4 ± 2	n.s.
Number of embedded social networks	2.1 ± 1	1.9 ± 1.2	n.s.
Family contacts			
Total number	6.1 ± 3.2	6.1 ± 3.4	n.s.
Diversity	3.3 ± 1.3	3 ± 1.3	n.s.
PQ-16			
Negative symptoms	0.3 ± 0.6	0.6 ± 0.7	*F* (1, 160) = 7.8, *p* < 0.01, *η*^2^ = 0.05
Unusual thought content, delusional ideas and paranoia	1.0 ± 1.1	1.2 ± 1.2	n.s.
Perceptual abnormalities and hallucinations	1.3 ± 1.8	1.9 ± 1.8	*F* (1, 160) = 4.5, *p* < 0.05, η^2^ = 0.03
Total	2.6 ± 2.2	4 ± 3.1	*F* (1, 160)=11.1, *p* < 0.005, *η*^2^ = 0.06
Total distress	2.3 ± 3.6	4.1 ± 4.6	*F* (1, 160) = 8, *p* = 0.01, *η*^2^ = 0.05
PQ-16 threshold			
(PQ-16 > 5 / PQ-16 < 6)	12/95	18/37	*χ*^2^(df = 1) = 11.1, *p* < 0.001

*Regular participants filled in all questionnaires; **Lost to follow-up participants filled in at least the first questionnaire; some of them also filled in one or two others (see details in the table). Statistics are based on *χ*^2^ or ANOVAs, as indicated in the table.

Then, we analyzed the longitudinal data by considering only those participants who filled in all questionnaires. It should be noted that when only one response was missing in a scale, we interpolated the data for this item. We also included the participants not writing any narrative. This allowed us to rescue the data in 13 participants (results were similar without those 13 participants), leading to the longitudinal analysis of 107 participants answering to all questionnaires.

### Participants who completed all four assessments (P-All)

**Fig. 1:**
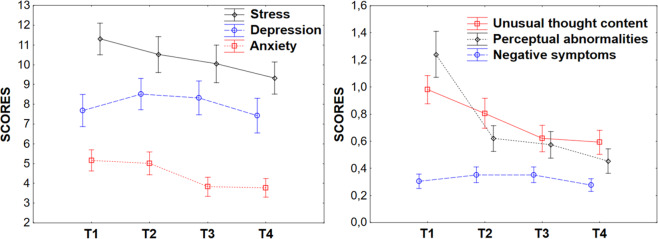
DASS-42 and PQ-16 scores. The DASS-42 scores (stress, depression, anxiety) across time are on the left panel, and the PQ-16 sub-scores (unusual thought content, perceptual abnormalities, and negative symptoms) on the right panel. Scores are averaged over participants and displayed with SEM.

We examined how clinical measures changed over time in the P-All group. Scores of both the DASS-42 and the PQ-16 were the highest on T1 (Fig. 1). They were largely correlated with each other on each measure (Table 3).

**Table 3:** Correlations between DASS-42 and PQ-16.

*N* = 107		T1	T2	T3	T4
		DASS-42	DASS-42	DASS-42	DASS-42
T1	PQ-16	*r* = 0.51**	*r* = 0.43**	*r* = 0.45**	*r* = 0.29*
	PQ-16 total distress	*r* = 0.51**	*r* = 0.44**	*r* = 0.45**	*r* = 0.47**
T2	PQ-16	*r* = 0.48**	*r* = 0.45**	*r* = 0.46**	*r* = 0.40**
	PQ-16 total distress	*r* = 0.45**	*r* = 0.46**	*r* = 0.49**	*r* = 0.46**
T3	PQ-16	*r* = 0.38**	*r* = 0.44**	*r* = 0.46**	*r* = 0.40**
	PQ-16 total distress	*r* = 0.38**	*r* = 0.48**	*r* = 0.55**	*r* = 0.47**
T4	PQ-16	*r* = 0.35**	*r* = 0.34**	*r* = 0.36**	*r* = 0.49**
	PQ-16 total distress	*r* = 0.30*	*r* = 0.39**	*r* = 0.41**	*r* = 0.52**

*p < 0.005; **p < 0.001 (Pearson correlations).

#### DASS-42

The repeated measures analyses of variance (ANOVA) on the DASS-42 scores using the three sub-scores (depression, anxiety, stress) and time (T1, T2, T3, T4) as within-subject variables showed no main effect of time (F[3, 318] = 1.8, n.s., partial *η*^2^=.01) but a significant interaction between time and DASS-42 sub-scores (*F*[6, 636] = 2.4, *p* < 0.05, partial *η*^2^ = 0.02). This was explained by the fact that anxiety decreased significantly across time (F[3, 318] = 4.2, *p* < 0.01, partial *η*^2^ = 0.04) whereas neither the depression nor the stress scores varied across time (Fs [3, 318]<2.1, n.s., partial *η*^2^ ≤ 0.02).

### PQ-16

The repeated measures ANOVA on the PQ-16 scores, with the three sub-scores and with time as within-subject variables, showed a main effect of time (averaged total scores decreased from 2.6 to 1.3, *F*[3, 318] = 17.5, *p* < 001), and a significant interaction between time and PQ-16 sub-scores (*F*(6, 636) = 10.3, *p* < 0.001, partial *η*^2^ = 0.088). The latter was explained by the fact that while the negative symptoms sub-score did not vary across time (*F*[3, 318] = 1.4, n.s., partial *η*^2^ = 0.01), the two other sub-scores significantly decreased across time (*F*[3, 318] = 10, *p* < 0.001, partial *η*^2^ = 0.09 for the unusual thought content, delusional ideas and paranoia sub-score, and *F*[3, 318] = 16.6, *p* < 0.001, partial *η*^2^ = 0.14 for the perceptual abnormalities and hallucinations sub-score). The total distress associated with PQ-16 symptoms also decreased across measures (taking the averaged values of 2.3, 1.4, 1.3 and 1.1 on successive measures, *F*[3, 318] = 15.8, *p* < 0.001, partial *η*^2^ = 0.13).

### Loneliness, social network, and concern regarding the pandemic

The feeling of loneliness did not vary significantly over time (*F*[3, 318] = 0.6, n.s., partial η^2^ = 0.0006). In contrast, the social network diversity significantly differed over time (*F*[3, 318] = 10.5, *p* < 0.001, partial η^2^=0.09). The HSD Tukey post-hoc analysis showed that the diversity of contact was higher on the mid-lockdown period (6.1 on T2, 6.0 on T3) than on the initial measure (5.6 on T1, keeping in mind that T1 participants were asked to refer to the period before lockdown) and after the lockdown (5.6 on T4), all ps < 0.001. The total number of contacts similarly evolved across time (*F*[3, 318] = 6.4, *p* < 0.001, partial *η*^2^ = 0.06). The HSD Tukey post-hoc test showed an increase of the number of contacts from T1 (17.5) to T2 (19.7, *p* < 0.001) which then remained stable. The number of social networks did not evolve across time (*F*[3, 318] = 1.2, n.s., partial *η*^2^ = 0.01).

The concern regarding the pandemic decreased with time (*F*[3, 318] = 22, *p* < 0.001, partial *η*^2^ = 0.17). The HSD Tukey post-hoc test showed that concern decreased between T1 (1.6) and T3 (1.3, *p* < 0.001), and decreased further in T4 (1.1, *p* < 0.01).

### Analyses of the diary narratives

Participants were asked to write down short narratives (about ten lines) of their lockdown subjective experience on a daily basis. Each separate diary entry was counted as one narrative essay. In general, more participants produced narratives at the beginning of the study, i.e. the second week of the lockdown period, than at any other time. Therefore, we focused on this second week.

Participants with high APS (PQ-16 > 5) produced fewer narratives on week 2 than those with low APS (F[1, 160]=4, *p* < 0.05, partial *η*^2^ = 0.024), but not fewer words per narration (a similar result is found when comparing the P-Lost and P-All groups, except there is additionally less words per narration on week 2; see Table 4).

**Table 4:** Characteristics of narratives of P-All and P-Lost at the beginning of lockdown.

	P-All* participants	P-Lost** participants	Statistical comparison of the scores between groups
Total number of narratives	39.9 ± 13.3	13.5 ± 14.3	*F* (1, 160) = 135.8, *p* < 0.001, *η*^2^ = 0.46
Total number of narratives during week 2	5.8 ± 1.7	3.8 ± 2.7	*F* (1, 160) = 24.1, *p* < 0.001, *η*^2^ = 0.13
Average number of words per narrative (subjects without narratives excluded)	207.4 ± 86.3	193 ± 104.7	*F* (1, 153) = 0.18, n.s. *η*^2^ = 0.001
Average number of words per narrative during week 2 (subjects without narratives excluded)	217 ± 88	176 ± 92	*F* (1, 148) = 5.6, *p* < 0.05 *η*^2^ = 0.036
Ratio of negative among emotional words in the narratives of week 2	51.6% ± 13%	52.5% ± 23%	*F* (1, 134) = 0.38, n.s. *η*^2^ = 0.003

Statistics are based on ANOVAs.

*P-All participants filled in all questionnaires; **P-Lost participants filled in the first questionnaire but did not complete all of them.

Given that the number of narratives (diary entries) produced and, to some extent, the average number of words per narrative varied between individuals, we calculated the ratio of negative emotion words among all emotional words (rather than per narrative). This ratio is less affected by the structural changes in the narratives. Exploratory correlation analyses among the participants who wrote narratives during week 2 showed that this ratio was positively correlated with the PQ-16 scores (*N* = 112; *r* = 0.22, *p* = 0.021) and with the total PQ-16 distress score (*N* = 112; r = 0.29, *p* = 0.002) at T2 (mid-lockdown measure). In contrast, the number of narratives (and not the mean number of words per narratives nor the ratio of emotional words) during week 2 was correlated positively with the DASS-42 score (*N* = 112; *r* = 0.24, *p* = 0.012). Results remained unchanged when analyses were restricted to the P-All.

Additionally, we calculated the average number of uses of the pronoun ‘I’ per narration on week 2 as it is known to be related to emotional distress^38^. In our results, the average number of uses of the pronoun ‘I’ was positively correlated with the total PQ-16 distress score (*N* = 112; *r* = 0.25, *p* = 0.008) and with the DASS-42 score (*N* = 112; *r* = 0.33, *p* < .001) at T2 (mid-lockdown measure).

### Clinical symptoms and psychosocial factors

We did not find a significant impact of age, town sizes, education levels, or gender on symptoms. Then, we explored correlations between clinical symptoms (PQ-16 and DASS-42) and psychosocial factors such as the degree of concern regarding the pandemic, feeling of loneliness, and social network contacts. We conducted correlations for each time of measure (T1, T2, T3, T4) in the 107 P-All (see Table 5). These analyses revealed that loneliness was correlated to the DASS-42 score at each time of measure, and at T1, T3, and T4 with the PQ-16 score. The social network index was correlated with the DASS-42. Social network measures were never correlated with the PQ-16 score. Loneliness and social network measures were correlated with each other at each time point (ps < 0.001, data not shown).

**Table 5:** Correlations between the DASS-42 and PQ-16 at the four time points, and various indices of social contacts, and concern regarding the COVID-19 pandemic on corresponding time points.

*N* = 107	Levels of concern regarding the pandemic	Loneliness	Social Network Index		
			Total number of contacts	Number of embedded networks	Diversity of contacts
T1 DASS-42	–	*r* = 0.31**	–	–	–
T1 PQ-16	–	*r* = 0.27*	–	–	–
T2 DASS-42	*r* = 0.29**	*r* = 0.25*	*r* = −0.32**	*r* = −0.32**	–
T2 PQ-16	–	–	–	–	–
T3 DASS-42	–	r = 0.31**	–	*r* = −0.25*	–
T3 PQ-16	–	*r* = 0.32**	–	–	–
T4 DASS-42	–	*r* = 0.35***	–	–	*r* = −0.27*
T4 PQ-16	–	*r* = 0.34**	–	–	–

*p < 0.01; **p < 0.005; ***p < 0.001 (Pearson correlations; we discarded correlations that did not survive the FDR correction).

## Discussion

The aim of our study was to examine the impact of the pandemic-induced lockdowns on mental health using both validated quantitative scales and diary narratives to better capture the emotional experience of the participants. The collection of mental health data started at the very beginning of the lockdown in France. We replicated previous studies showing abnormal levels of psychological symptoms on the beginning of the study and a decrease of symptoms across time, especially anxiety. Our study extends those results to attenuated psychotic symptoms. Several correlations suggest that our data is reliable, despite the limits of online screening questionnaires: first, we replicated previously reported correlations between symptoms of stress, anxiety and depression (the DASS-42) and several indexes of social contact and the feeling of loneliness. Second, APS are associated with APS-related distress, suggesting they represent true symptoms. The fact that APS are correlated with loneliness, DASS-42 scores, and negative emotions in the narratives further validates the results.

Several studies indicated that the beginning of the pandemic and global lockdown were followed by increased levels of stress, anxiety and depression in the general population in several European countries^3,7,23,39–41^. Longitudinal studies assessing the evolution of these symptoms during the first months of the pandemic (from March/April to May/August) showed mixed results. On the one hand, levels of stress, anxiety, and depression often decreased overtime, including in a large-scale survey with over 36,000 participants in the UK^11,42–44^. On the other hand, reports showed either stable or increasing symptoms of depression during the same period^10,42^. Consistent with our findings, the feeling of loneliness remained stable during this period in most reports^42,45^. Groarke et al.^46^ found a longitudinal association between depression and the feeling of loneliness in 1958 UK adults, while Novotny and al. found that loneliness was associated with an increase in stress levels and the severity of depression in 715 Czechs^41,46^. In Spain, loneliness was the main predictor of anxiety and depression^47^. In parallel, in the UK, daily face-to-face or phone/video contacts were associated with lower depression^48^. In a multinational study in France, USA, Korea, and Hong Kong, the most consistent effect was a link between psychological distress and the feeling of loneliness^4^. These results altogether show that despite our population specificities, mostly composed of highly educated women, with a stable job, the evolution of general mental health over time and its correlation with psychosocial factors were comparable with the general population.

To our knowledge, very few studies have explored the impact of the pandemic and lockdown on psychotic symptoms. Our results showed that 30 out of our 162 participants (18.5%) met the criteria for high-risk at the beginning of the lockdown. This prevalence is twice that of 9.3% reported in previous studies conducted in non-help-seeking individuals^49^. It is worth reminding that none of the participants who finished the study endorsed the APS criteria at the end of the study. This means that APS observed at the beginning of our study were not related to a psychosis proneness of our participants (at least in those who completed the study) but rather, indicate psychotic symptoms temporarily elevated by stressful events. We can expect that at T1, participants were influenced by their pre-lockdown memories as well as their current state when filling in the questionnaires, and these combined, stress-provoking influences may have led to increased APS at the beginning of the study. Looking more precisely at the kind of APS, abnormal perceptions, and unusual thought contents were the dimensions associated with the highest scores on T1, which later decreased significantly over time.

Our results align with those of a previous study showing an increase of perceptual disturbances (mainly derealization and depersonalization), subclinical psychotic symptoms, and beliefs in the pseudoscience after the lockdown^50^. Another study found that the presence of hallucinations and paranoia was observed only in people with negative affect (fear of COVID-19) and low political trust, the exposure to COVID-19 news being a critical mediating factor between negative emotions and psychotic symptoms^51^. In contrast, another study investigating a sample of 728 French subjects from the general population did not report increased scores of hallucinations and paranoid ideations (in comparison to scores generally reported in the general population)^26^. This study started one month after the beginning of the lockdown (corresponding to T2 of our study) and may thus have failed to capture early elevated APS.

As expected, we found a significant correlation between loneliness and APS, at least in T1, T3, and T4. Yet several results suggest that negative emotions may have played a larger role than loneliness in the emergence of APS on T1. Loneliness did not vary across time, whereas APS decreased along with the distress associated to the APS. APS were related to the symptoms of depression, anxiety, and stress. Moreover, the link between APS and emotions is supported by the results of narratives. We show that variations in narratives, particularly the use of negative emotional words, were associated with attenuated psychotic symptoms. These observations are important because they tend to support the validity of the APS results. The validity of the results is further reinforced by the observation of a link between the use of pronoun ‘I’, the DASS-42 scores, and also the distress associated to APS. A link between the increased use of the pronoun ‘I’ and mental illness has already been reported in the literature^52–55^ and is considered as an index of emotional distress^38^.

The link between APS and emotions is in line with stress-reactivity hypotheses in psychosis which state that aberrant emotional reactivity to daily (or unusual) stress supports an affective pathway to psychosis^1^. Moreover, this model states that affective pathways are independent of cognitive impairments and lead to more episodic and good-outcome types of psychosis, constituting the dimensions explored by the PQ-16.

In line with our results, Najolia et al. found an affective dysfunction in schizotypy^56^. They found that schizotypy was associated with less positive and more negative words in verbal reactions to emotional stimuli, particularly for pleasant stimuli. Writing about personal experiences in an emotional way has been shown to enhance mental and physical health^57,58^. Individuals who most beneficiate from their written narratives tend to use higher positive-emotion words and few negative emotion words^58^. In the present study, the large proportion of negative words among emotional words in participants with APS may all the more reveal their ill-being^59^. On the other hand, participants with higher scores of depressive, stress, and anxiety symptoms (measured with DASS-42) wrote more narratives, but did not show the same association with negative words. Sharing emotional experiences through storytelling plays a central role in emotion process and regulation^60^. Indeed, narrating emotional events serves a cathartic effect of getting rid of negative emotions^61^ and also creates a space for cognitive understanding and appraisal of the event by organizing it in a causal-motivational sequence^58^. Writing narratives is also an act of sharing one’s own subjective experience with others and support social belongingness^62^. Hence, participants with more depressive and anxiety symptoms might have used the written daily narratives as a way to regulate emotions associated with the pandemic and the lockdown, allowing them to feel integrated in a common social experience.

In addition to the link between negative emotion and APS, our results raise several additional questions. Among those with high APS, two-thirds belonged to the group that did not fill in all questionnaires although their stress, anxiety, and depression levels did not differ from those who completed the surveys. Those who filled in 3 questionnaires had decreasing symptoms over time, which suggests that results are robust. Nonetheless 8 participants among 30 reaching the cut-off score of APS dropped out after T1 and therefore, their outcomes are unknown. They also wrote fewer narratives, and as APS level was associated with more negatively-toned narrations, it seems likely that those people are the most in need for psychological support. As for the evolution of the symptoms over time, it is like a two-sided coin. On one hand, one can emphasize the resilience of our participants by pointing to the decrease of symptoms over time and the relatively low number of participants stopping after T1. However, this was only the first lockdown period, our participants were privileged and well-educated, and they managed to increase their social contacts during the lockdown period. Despite these protective conditions, APS increased at T1. These results suggest that vulnerability to APS is shared by many and can therefore lead to psychotic symptoms when anxiety-provoking situations arise. Therefore, this suggests that questioning APS symptoms should not be a taboo.

Our study has some limitations. First, our sample is relatively small, which was intended to preserve feasibility, as life narratives analyses are time-consuming. Secondly, our sample is not representative of the general population as it is mainly composed of highly educated women. It had the advantage of preserving homogeneity and focus on a community of highly educated stable teleworkers. As in the case of most online surveys, our study is based on self-reports and cannot offer any clinical evaluation of the subjects; similarly, even though only a handful of participants reported having been ill from COVID, none having reported substance dependence, this information could not be independently verified. Although the automatic analysis of the narratives provided partial validation of the results, the large volume of narratives collected during the study (>5000) prevented us from performing more fine-grained analyses targeting self-disturbance-related issues^63^.

In conclusion, our study showed that in a sample of highly educated individuals, the psychological impact of the first lockdown during COVID-19 pandemic unfolded similarly to the general population worldwide. Moreover, we highlighted the emergence of attenuated psychotic symptoms and its correlation with stress, anxiety, depression, as well as negative emotional words in the daily narratives, and an increase of the use of the personal pronoun “I” which replicates data observed in psychosis. The results show both a vulnerability in the general community (the results in T1) and a resilience (the decrease of symptoms over time). The fact that many participants experience APS, even if transiently, highlights the existing continuum between psychosis and the general community and emphasizes the importance of screening and addressing APS during this crisis period.

## Methods

### Participants and procedure

A longitudinal design was chosen to assess the evolution of mental health during and after lockdown in the general community. The study advertisement was distributed by e-mail among French researcher networks (University of Strasbourg, INSERM, and CNRS) before being relayed more widely in France. The study protocol and informed consent procedure were approved by the ethical committee of the University of Strasbourg (Unistra/CER/2020-10) and were in accordance with the Declaration of Helsinki. The informed consent to participate was signed and transmitted online to the PI of the study. Thereafter, the privacy of the participants was completely protected by creating individual pseudonyms. Participants used the pseudonym to identify any document they uploaded on a secured online storage system (Seafile^®^) shared with investigators and hosted by the University of Strasbourg.

The first COVID-19 lockdown began in France on March 17, 2020 and ended on May 11, 2020. During this period, the participants completed self-report questionnaires at three different periods: T1 (*lockdown beginning*, filled in between March 19 and March 31); T2 (*middle of lockdown*, filled in between April 17 and April 27) and T3 (*end of lockdown*, filled in between May 8 and May 22). They also filled in self-report questionnaires after the lockdown period: T4 (*after lockdown*, filled in between June 22 and July 18). We obtained an ethical agreement on March 20, and were able to include participants at the end of the first week of lockdown. Given the close temporal proximity with the period preceding the lockdown, in T1 participants were asked to fill in the questionnaires by referring to their pre-lockdown state. T1 is referred to as the beginning of lockdown. Concurrently, participants were asked to write regularly about their daily life during the lockdown period. Their narratives and answers to the questionnaires were collected on Seafile^®^.

### Sociodemographic data and living conditions

Sociodemographic data were collected on age, gender, employment status, educational level, and marital status. The participants self-assessed their level of concern about COVID-19 (no concern, minor, moderate, or major concern). We collected data on the following lockdown conditions: household type (apartment, house) and size, and access to nature or not. Participants specified whether they were working from home or not.

### Questionnaires about mental health and social contacts

Mental health status was measured using the Depression, Anxiety, and Stress Scale (DASS-42). Questions 3, 5, 10, 13, 16, 17, and 21 formed the depression subscale. Questions 2, 4, 7, 9, 15, 19, and 20 formed the anxiety subscale. Questions 1, 6, 8, 11, 12, 14, and 18 formed the stress subscale.

The PQ-16 questionnaire was used to measure APS. Each item is scored as present (1) or absent (0), and the sum yields the PQ-16 total score. We used the typical cut-off score of >5 to differentiate participants with or without significant attenuated psychosis symptoms. Items 1 and 7 formed the ‘negative symptoms’ subscale. Items 2, 5, 10, 11, and 14 formed the ‘unusual thought content’ subscale, while items 3, 4, 6, 8, 9, 12, 13, 15, and 16 formed the ‘perceptual abnormalities’ sub-score.

Each item is rated on a 4-point Likert scale assessing the distress related to each symptom (from 0 = none to 3 = severe). A total score ranging from 0 to 48 was calculated.

### Daily life narratives

The profile of participants’ emotional experiences was assessed using EMOTAIX^©^ emotional lexicon^64^, under Tropes^©^ (version 8.5) software, to automatically identify, categorize, and count the total number of words, the number of emotional (positive and negative) words, and the number and type of pronouns.

### Statistical analysis

Separate ANOVA for repeated measures were conducted on mental health scores with time (T1, T2, T3, T4) as a within-subject variable. Post-hoc analyses were conducted using Tukey HSD. The association between mental health scores and living conditions was explored using correlation analyses based on the false discovery rate (FDR) method, a rigorous method of alpha-level adjustment for multiple comparisons^65^. Statistical analyses were performed using SPSS Statistic 21.0 (IBM SPSS Statistics, New York, United States) and JASP 0.10.1 (JASP Team, Netherlands).

### Reporting summary

Further information on research design is available in the Nature Research Reporting Summary linked to this article.

## Supplementary information

REPORTING SUMMARY

## Data Availability

The datasets analyzed during the current study are available from the corresponding author upon reasonable request.
