# Supplementary material for: Facing the pandemic and lockdown: an insight on mental health from a longitudinal study using diaries
Source: Schizophrenia (Heidelb). 2022 Mar 15;8(1):22. doi: 10.1038/s41537-022-00222-2 (PMC8922066; doi:10.1038/s41537-022-00222-2)
Supplement: Supplementary file 1 — REPORTING SUMMARY [file 41537_2022_222_MOESM1_ESM.pdf]

nature portfolio

Corresponding author(s): Anne GIERSCH

Last updated by author(s): Oct 5, 2021

Reporting Summary

Nature Portfolio wishes to improve the reproducibility of the work that we publish. This form provides structure for consistency and transparency in reporting. For further information on Nature Portfolio policies, see our Editorial Policies and the Editorial Policy Checklist.

Statistics

For all statistical analyses, confirm that the following items are present in the figure legend, table legend, main text, or Methods section.

n/a	Confirmed	
☐	☒	The exact sample size (n) for each experimental group/condition, given as a discrete number and unit of measurement
☐	☒	A statement on whether measurements were taken from distinct samples or whether the same sample was measured repeatedly
☐	☒	The statistical test(s) used AND whether they are one- or two-sided<br>*Only common tests should be described solely by name; describe more complex techniques in the Methods section.*
☒	☐	A description of all covariates tested
☐	☒	A description of any assumptions or corrections, such as tests of normality and adjustment for multiple comparisons
☐	☒	A full description of the statistical parameters including central tendency (e.g. means) or other basic estimates (e.g. regression coefficient) AND variation (e.g. standard deviation) or associated estimates of uncertainty (e.g. confidence intervals)
☐	☒	For null hypothesis testing, the test statistic (e.g. F, t, r) with confidence intervals, effect sizes, degrees of freedom and P value noted<br>*Give P values as exact values whenever suitable.*
☒	☐	For Bayesian analysis, information on the choice of priors and Markov chain Monte Carlo settings
☐	☒	For hierarchical and complex designs, identification of the appropriate level for tests and full reporting of outcomes
☐	☒	Estimates of effect sizes (e.g. Cohen's d, Pearson's r), indicating how they were calculated

Our web collection on statistics for biologists contains articles on many of the points above.

Software and code

Policy information about availability of computer code

Data collection	LimeSurvey version 2
Data analysis	SPSS Statistic 21.0, JASP 0.10.1, for the narrative analyses: TROPES version 8.5 and EMOTAIX

For manuscripts utilizing custom algorithms or software that are central to the research but not yet described in published literature, software must be made available to editors and reviewers. We strongly encourage code deposition in a community repository (e.g. GitHub). See the Nature Portfolio guidelines for submitting code & software for further information.

Data

Policy information about availability of data

All manuscripts must include a data availability statement. This statement should provide the following information, where applicable:

- Accession codes, unique identifiers, or web links for publicly available datasets
- A description of any restrictions on data availability
- For clinical datasets or third party data, please ensure that the statement adheres to our policy

The datasets analyzed during the current study are available from the corresponding author upon reasonable request.

Field-specific reporting

Please select the one below that is the best fit for your research. If you are not sure, read the appropriate sections before making your selection.

☐ Life sciences ☒ Behavioural & social sciences ☐ Ecological, evolutionary & environmental sciences

For a reference copy of the document with all sections, see nature.com/documents/nr-reporting-summary-flat.pdf

Behavioural & social sciences study design

All studies must disclose on these points even when the disclosure is negative.

Study description	Participants (N=162) completed an online survey 4 times between March and June 2020 (T1,T2,T3,T4). Depression, anxiety, and stress were self-assessed with the DASS-42 and APS with the PQ-16, which were provided online. Psychosocial data such as the feeling of loneliness were also assessed. The participants wrote daily narratives during the lockdown period.
Research sample	162 participants were recruited (mean age 42.9 SD 14.8; 80% of women; the vast majority of the participants were highly educated). The sample is not representative of the general population and this is made clear in the manuscript. This was justified by our wish to collect daily narratives from the participants. The fact that we nonetheless observe attenuated psychosis symptoms inT1 is noteworthy.
Sampling strategy	This was one unique sample of participants. The calculation of the sample size was impossible, given we had no prior data on the effect of the pandemy or lockdown at the time. Our study is not epidemiological, and the sample size is a compromise between the hope to collect as many data as possible, the knowledge that collecting narratives would bias our sample, and our wish to collect data as soon as possible after the lockdown beginning, which led us to stop inclusions 2 weeks after the lockdown beginning .
Data collection	We provided the questionnaires to the participants through internet, and we used Lime-Survey to collect the data. The narrations were written on a text software and transformed in pdf by the participants, who deposited them anonymously on the cloud of the University of Strasbourg
Timing	The first COVID-19 lockdown began in France on March 17, 2020, and ended on May 11, 2020. During this period, the participants completed self-report questionnaires at three different periods: T1 (lockdown beginning, filled in between March 19 and March 31); T2 (middle of lockdown, filled in between April 17 and April 27) and T3 (end of lockdown, filled in between May 8 and May 22). They also filled self-report questionnaires after the lockdown period: T4 (after lockdown, filled in between June 22 and July 18).
Data exclusions	no data was excluded
Non-participation	Among the 162 participants, 107 participants filled in all questionnaires, and 55 dropped out. The time when they dropped out is detailed in the manuscript, and the analysis showed that the symptoms displayed by the participants dropping out differed on T1 from the 107 participants filling in all questionnaires
Randomization	The participants were not allocated in experimental groups

Reporting for specific materials, systems and methods

We require information from authors about some types of materials, experimental systems and methods used in many studies. Here, indicate whether each material, system or method listed is relevant to your study. If you are not sure if a list item applies to your research, read the appropriate section before selecting a response.

Materials & experimental systems

n/a	Involved in the study	
☒	☐	Antibodies
☒	☐	Eukaryotic cell lines
☒	☐	Palaeontology and archaeology
☒	☐	Animals and other organisms
☐	☒	Human research participants
☒	☐	Clinical data
☒	☐	Dual use research of concern

Methods

n/a	Involved in the study	
☒	☐	ChIP-seq
☒	☐	Flow cytometry
☒	☐	MRI-based neuroimaging

Human research participants

Policy information about studies involving human research participants

Population characteristics	General population contacted through social networks during the first lockdown in France

Recruitment	The study advertisement was distributed by e-mail, among French researcher networks (University of Strasbourg, INSERM and CNRS), and were then relayed more widely in France. It may explained that the sample is composed of highly educated individuals, as well as the instructions to write daily narratives. This is developed in the manuscript. The informed consent to participate was signed and transmitted online to the PI of the study. Thereafter the privacy of the participants was completely protected by creating individual pseudonyms. Participants used the pseudonym to identify any document they uploaded on a secured online storage system (Seafile®) shared with investigators and hosted by the University of Strasbourg.
Ethics oversight	Ethical committee of the University of Strasbourg Unistra/CER/2020-10

Note that full information on the approval of the study protocol must also be provided in the manuscript.
